# Circulating and Adipose Tissue miRNAs in Women With Polycystic Ovary Syndrome and Responses to High-Intensity Interval Training

**DOI:** 10.3389/fphys.2020.00904

**Published:** 2020-07-30

**Authors:** Sofie Lionett, Ida A. Kiel, Donny M. Camera, Eszter Vanky, Evelyn B. Parr, Stian Lydersen, John A. Hawley, Trine Moholdt

**Affiliations:** ^1^Department of Circulation and Medical Imaging, Faculty of Medicine and Health Sciences, Norwegian University of Science and Technology, Trondheim, Norway; ^2^Department of Obstetrics and Gynecology, St. Olav’s Hospital, Trondheim, Norway; ^3^Exercise and Nutrition Research Program, Mary MacKillop Institute for Health Research, Australian Catholic University, Melbourne, VIC, Australia; ^4^Department of Health and Medical Sciences, Swinburne University of Technology, Melbourne, VIC, Australia; ^5^Regional Centre for Child and Youth Mental Health and Child Welfare, Norwegian University of Science and Technology, Trondheim, Norway

**Keywords:** exercise, miRNA-27b, insulin resistance, epigenetic modifications, cardiorespiratory fitness, female

## Abstract

MicroRNAs (miRNAs) are small non-coding RNAs that regulate gene expression post-transcriptionally. In women with polycystic ovary syndrome (PCOS), several miRNAs are differentially expressed compared to women without PCOS, suggesting a role for miRNAs in PCOS pathophysiology. Exercise training modulates miRNA abundance and is primary lifestyle intervention for women with PCOS. Accordingly, we measured the expression of eight circulating miRNAs selected *a priori* along with miRNA expression from gluteal and abdominal adipose tissue (AT) in 12 women with PCOS and 12 women matched for age and body mass index without PCOS. We also determined the miRNA expression “signatures” before and after high-intensity interval training (HIT) in 42 women with PCOS randomized to either: (1) low-volume HIT (LV-HIT, 10 × 1 min work bouts at maximal, sustainable intensity, *n* = 13); (2) high-volume HIT (HV-HIT, 4 × 4 min work bouts reaching 90–95% of maximal heart rate, *n* = 14); or (3) non-exercise control (Non-Ex, *n* = 15). Both HIT groups trained three times/week for 16 weeks. miRNAs were extracted from plasma, gluteal and abdominal AT, and quantified via a customized plate array containing eight miRNAs associated with PCOS and/or exercise training responses. Basal expression of circulating miRNA-27b (c-miR-27b), implicated in fatty acid metabolism, adipocyte differentiation and inflammation, was 1.8-fold higher in women with compared to without PCOS (*P* = 0.006) despite no difference in gluteal or abdominal AT miR-27b expression. Only the HV-HIT protocol increased peak oxygen uptake (VO_2_peak L/min; 9%, *P* = 0.008). There were no changes in body composition. In LV-HIT, but not HV-HIT, the expression of c-miR-27b decreased (0.5-fold, *P* = 0.007). None of the remaining seven circulating miRNAs changed in LV-HIT, nor was the expression of gluteal or abdominal AT miRNAs altered. Despite increased cardiorespiratory fitness, HV-HIT did not alter the expression of any circulating, gluteal or abdominal AT miRNAs. We conclude that women with PCOS have a higher basal expression of c-miR-27b compared to women without PCOS and that 16 weeks of LV-HIT reduces the expression of this miRNA in women with PCOS. Intense exercise training had little effect on the abundance of the selected miRNAs within subcutaneous AT depots in women with PCOS.

## Introduction

Polycystic ovary syndrome (PCOS) is the most common endocrine disorder in reproductive-aged women, affecting up to 13% of females globally (Bozdag et al., 2016). Beyond causing infertility (Teede et al., 2011), PCOS is associated with whole-body insulin resistance and metabolic disorders such as obesity and type 2 diabetes mellitus (Anderson et al., 2014; Cassar et al., 2016; Orio et al., 2016). Despite the high prevalence and adverse health implications of PCOS, the etiology and optimal treatments for women with PCOS are unclear. Insulin resistance is an underlying feature of PCOS (Teede et al., 2011). Indeed, insulin sensitivity is 27% lower in women with PCOS compared to healthy controls (Cassar et al., 2016), with up to 85% of women with PCOS being insulin resistant (Stepto et al., 2013). Lifestyle interventions, including exercise training, are recommended as first-line therapy for women with PCOS (Teede et al., 2011, 2018). In this regard, a growing body of evidence demonstrate that high-intensity interval training (HIT) confers greater health benefits compared to moderate intensity exercise in clinical cohorts (Weston et al., 2014; Cassidy et al., 2017). Such superior effects of vigorous intensity exercise are also evident among women with PCOS (Patten et al., 2020). Thus, a greater knowledge of the molecular mechanisms underlying the efficacy of exercise interventions such as HIT on metabolic health may help to understand the pathophysiology of PCOS.

MicroRNAs (miRNAs) are small non-coding RNAs that act as post-transcriptional regulators by binding with specific mRNA transcripts and preventing protein and gene translation or by degrading the target mRNA (Chen X. et al., 2012). miRNAs can function within the tissue of origin or adjacent tissues (Chen X. et al., 2012), thus playing crucial regulatory roles in many biological processes. Several studies in women with PCOS have reported altered expression of miRNAs in the circulation (Murri et al., 2013, 2018; Long et al., 2014; Sorensen et al., 2014, 2019; Ding et al., 2015; Sathyapalan et al., 2015; Arancio et al., 2018; Chen et al., 2019), adipose tissue (AT) (Chen et al., 2013, 2019; Sorensen et al., 2014; Chuang et al., 2015), and follicular fluid and granulosa cells (Sorensen et al., 2014; Butler et al., 2019; Chen et al., 2019), suggesting a potential role for miRNAs in the pathophysiology of this condition. Specifically, abnormal expression of miRNAs with putative roles in regulating glucose metabolism in adipocytes in insulin resistant women with PCOS have been observed (Chen et al., 2013; Wu et al., 2014; Chuang et al., 2015). Furthermore, studies have reported significantly abnormal expression of miRNAs in subcutaneous abdominal AT in insulin resistant women, regardless of PCOS status (Wu et al., 2014; Chuang et al., 2015).

Women with PCOS have larger adipocytes and reduced adipocyte insulin sensitivity compared to women matched for body mass index (BMI) without PCOS (Dunaif et al., 1992; Manneras-Holm et al., 2011). As such, miRNAs could play a role in these AT abnormalities in women with PCOS. Abdominal obesity is associated with increased risk of insulin resistance and cardiovascular disease (Karpe and Pinnick, 2015) and women with PCOS have greater abdominal fat (Fauser et al., 2012). AT from different depots express unique molecular, cellular and metabolic properties and respond in distinct ways to exercise and nutritional challenges (Tan et al., 2004; You et al., 2014; Tsiloulis et al., 2017). Therefore, in order to understand these differential expression profiles, interrogation of miRNAs from different AT depots in women with PCOS is necessary. However, to the best of our knowledge, such profiles are currently lacking. Accordingly, the primary aim of the present investigation was to compare the expression of selected miRNAs in the circulation, gluteal and abdominal AT in women with PCOS to an age- and BMI-matched control group of women without PCOS. Secondary aims were to investigate the effects of two chronic protocols of HIT on the “expression signature” of these miRNAs in women with PCOS, and to assess differences in the selected miRNA expressions in gluteal and abdominal AT and whether the two AT depots responded in distinct ways to HIT. Our main hypotheses were that there would be differential expression patterns in circulating and AT miRNA’s in women with, compared to those without PCOS, and that the expression signature would be modulated by chronic exercise training in women with PCOS.

## Materials and Methods

### Participants and Study Design

Forty-two women with PCOS and 12 women without PCOS (Non-PCOS) were recruited for this study, which was part of the Improving Reproductive Function in Women with Polycystic Ovary Syndrome with High-Intensity Interval Training (IMPROV-IT) trial (Kiel et al., 2020) (ClinicalTrials.gov Identifier: NCT02419482) and of the Adipose Tissue Function and Response to Exercise Training in Women With and Without Polycystic Ovary Syndrome trial (HIT-FAT; ClinicalTrials.gov Identifier NCT02943291). The IMPROV-IT trial is a two-center randomized controlled trial undertaken at The Norwegian University of Science and Technology (NTNU) in Trondheim, Norway and at The Australian Catholic University (ACU) in Melbourne, Australia. The study protocol for the IMPROV-IT trial has been published previously (Kiel et al., 2020). Non-PCOS participants were recruited only in Norway. We selected 12 women without PCOS (Non-PCOS) as a control group, who were individually matched to 12 women with PCOS. Women with and without PCOS were individually matched by age and BMI with an age difference of 5 years and BMI difference within 2 kg/m^2^. PCOS was defined according to the Rotterdam criteria, where a minimum of two of the following three conditions must be present: (1) polycystic ovary morphology (12 or more 2–9 mm follicles or >10 mL in volume in at least one ovary), (2) hyperandrogenism (either clinical signs such as acne or hirsutism, or biomedical) and/or oligo/amenorrhea (Rotterdam, 2004). Hirsutism was defined with a Ferriman Gallwey score ≥8 (Ferriman and Gallwey, 1961). The cut-off values for biochemical hyperandrogenism were defined as a testosterone concentration of >3.0 nmol/L, calculated free testosterone concentration of >32 pmol/L, sex hormone binding globulin (SHBG) concentration of <30 nmol/L, or free androgen index (FAI as 100× testosterone concentration (nmol/L)/SHBG concentration (nmol/L) >5% (Al Kindi et al., 2012). Oligo/amenorrhea was defined as an intermenstrual interval >35 days and ≤9 menstruations in the past year. Amenorrhea was defined as absent menstruations in the past 90 days.

To be eligible for inclusion into the study, women had to be aged between 18 and 45 years. Exclusion criteria were; if women were undertaking regular endurance training ≥2 sessions/week, cardiovascular diseases or other endocrine disorders, pregnancy or breastfeeding within the last 24 weeks, physical ailments or injuries that would hinder exercise performance, or undergoing concurrent treatments with hormonal contraceptives, insulin sensitizers or drugs known to affect gonadotropin or ovulation (with a wash-out period of 3 months prior to inclusion). Women were not excluded based on dietary intake and were encouraged to continue with their habitual diet during the study period. For women without PCOS, inclusion and exclusion criteria were the same as for women with PCOS, but they were normally menstruating women with no evidence of hyperandrogenism or polycystic ovaries.

### Ethical Approval

The study was performed according to the Helsinki declaration and approved by The Regional Committee for Medical and Health Research Ethics in Central Norway (REK-midt 2015/468 and 2016/545) and the ACU Human Research Ethics Committee (2017-260H). Participants were informed about the experiments and potential risks verbally and their written consent was obtained prior to study entry.

### Pre- and Post-intervention Testing

**FIGURE 1:**
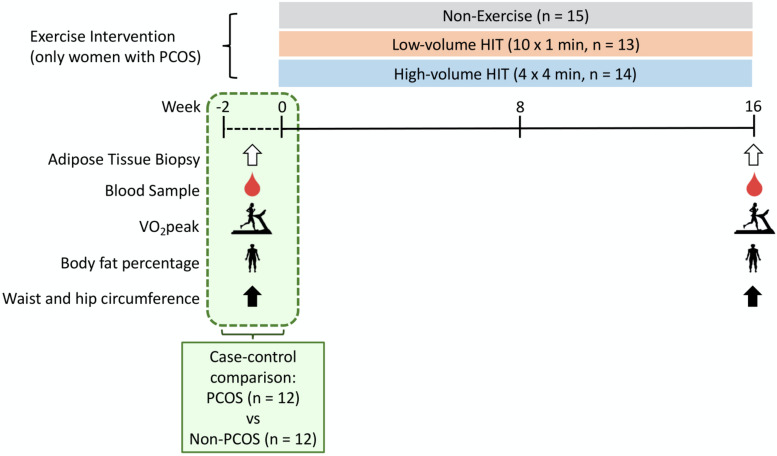
Study protocol. The testing days included: VO_2_peak, body fat percentage, waist and hip circumference measurements as well as blood and adipose tissue sampling. Non-PCOS (*n* = 12) women were only tested at baseline, whereas all women with PCOS (*n* = 42) were tested at baseline and after the 16 weeks exercise intervention. The 12 PCOS women in the case-control comparison also took part in the exercise intervention. Non-PCOS were age- and BMI-matched to these 12 women with PCOS.

Figure 1 displays an overview of the experimental protocol. Participants with a regular menstrual cycle were tested during the early follicular phase (day 1–7 of the menstrual cycle) whereas women with oligo/amenorrhea were tested independent of the time of their cycle. Participants performed an incremental test to exhaustion on a treadmill to measure peak oxygen uptake (VO_2_peak) and estimate maximal heart rate (HR_max_) using an individualized protocol. Following a 10 min warm-up and 3 min at moderate-intensity, the treadmill speed or incline was increased every 1–2 min by 0.5-1.0 km/h or 1–2% until volitional fatigue. After an overnight fast (≥12 h) and refraining from exercise for ≥48 h, participants returned to the laboratory and body fat percentage (BF%) was estimated. In Australia, the BF% was estimated using dual-energy X-ray absorptiometry (DXA; GE Lunar iDXA Pro, Encore software version 16, General Electric, Boston, MA, United States). In Norway, the BF% was estimated using bioelectrical impedance analysis (InBody720, Biospace CO, South Korea). Waist and hip circumference were also measured to the nearest 0.5 cm in duplicate. A resting blood sample was collected in a 4 mL EDTA and a 5 mL serum tube from the antecubital vein. The EDTA tube was immediately spun at 2,200 rpm at 20°C for 10 min while the serum tube rested 30 min before it was spun with the same protocol. Plasma was collected and stored at −80°C for subsequent analysis. On the same day, abdominal and gluteal subcutaneous AT biopsies (300–500 mg) were collected using a 14-gauge needle under local anesthesia with 1% xylocaine and washed on gauze with saline. Blood and connective tissue were removed before approximately 100 mg was snap-frozen in liquid nitrogen and stored at −80°C for subsequent miRNA analyses. Non-PCOS women were tested for all the measures described at baseline, whereas all women with PCOS were tested at baseline and after the 16 weeks exercise intervention (Figure 1). To avoid any residual effects of the last exercise session on miRNA expression, resting plasma and AT were sampled ≥48 h following the last exercise session.

### Training Protocols

Women with PCOS (*n* = 42) were randomized to 16 weeks of HIT or a non-exercising control group. Participants were stratified for a BMI < or ≥27 kg/m^2^ and randomly assigned in a 1:1:1 manner to one of three groups: (1) LV-HIT (*n* = 13); (2) HV-HIT (*n* = 14); (3) Non-exercise (Non-Ex, *n* = 15). The training program has been described previously (Kiel et al., 2020). Briefly, exercise training was performed on a treadmill or as outdoor running/walking three times/week. Women assigned to the LV-HIT group completed 10 × 1 min work bouts at the maximal intensity they could sustain, separated by 1 min of passive recovery or low-intensity walking. Women in the HV-HIT group completed 4 × 4 min work bouts reaching 90–95% HR_max_ during the first two minutes of each bout, separated by 3 min of active recovery at ∼70% of HR_max_. All training sessions included a 10 min warm-up and a 3-min cool-down period. At least one weekly session was supervised at the laboratory, and the participants wore HR monitors (Polar M400) during all sessions to ensure compliance with both exercise protocols. Women in the Non-Ex group were advised to continue their habitual physical activity and informed about the current recommendations for physical activity in adults.

### Circulating miRNA Extraction and Reverse Transcription

After centrifuging thawed plasma samples for 10 min at 4°C in order to remove cellular debris, circulating miRNAs (c-miRs) were extracted using the miRNeasy Serum/Plasma Advanced Kit (217204; Qiagen, Australia), which allows for extraction and purification of small (<200 nt) cell-free RNA. A total of 200 μL supernatant was then transferred to a new sterile tube. To assist with determining the yield of template recovered, 3.5 μL of a miRNeasy Serum/Plasma Spike-In Control (219610; Qiagen, Australia) was added to all samples prior to extraction of RNA. Input amounts of RNA were standardized using the same initial volume (2 μL) for all samples following the manufacturer’s instructions. RNA was reverse transcribed into cDNA by using the miRCURY LNA RT kit (339340; Qiagen, Australia) in a BioRad thermal cycler (BioRad, Australia). The resulting cDNA was stored at −20°C.

### Adipose Tissue miRNA Extraction and Reverse Transcription

MicroRNAs were extracted from frozen abdominal and gluteal AT samples using the miRNeasy Mini (217004; Qiagen, Australia) and RNeasy MinElute Cleanup (74204; Qiagen, Melbourne, VIC, Australia) Kits according to manufacturer’s instructions. Approximately 100 mg of AT was homogenized in QIAzol Lysis Reagent and phase separated with chloroform. Samples were then loaded into spin columns, washed with ethanol and eluted in 14 μL of RNAse-free water. Extracted miRNAs were quantified on a Qubit 4 Fluorometer using the Qubit microRNA Assay Kit (Q32881; Life Technologies, Australia). Samples were then equilibrated with RNase-free water and reverse transcribed to cDNA using the miRCURY LNA RT Kit (339340; Qiagen, Australia) in a BioRad thermal cycler (BioRad, Australia). The resulting cDNA was stored at −20°C.

### Targeted MicroRNA Quantification

Quantification of both circulating and AT miRNAs were performed on a Qiagen customized 96-well miScript miRNA PCR Array (339330; Qiagen, Australia) with miScript SYBR using a BioRad CFX96 (BioRad, Australia) following manufacturer’s instructions. The array contained eight miRNAs selected *a priori* based on previous reports of altered c-miRs profiles in women with PCOS and/or in response to exercise training (Murri et al., 2013; Sorensen et al., 2014; Improta et al., 2018; Da Silva et al., 2019). The selected c-miRs were hsa-miR-21-5p, hsa-miR-27b-5p, hsa-miR-93-5p, hsa-miR-146a-5p, hsa-miR-155-5p, hsa-miR-222-3p, hsa-miR-223-3p, and hsa-miR-103a-3p. These miRNAs have been shown to be implicated in hormone secretion and metabolism, inflammation, adipogenesis, and lipid and glucose metabolism (Chen W. et al., 2012; Murri et al., 2013; Sorensen et al., 2014; Chuang et al., 2015). The same eight miRNAs were investigated in the subcutaneous abdominal and gluteal AT (*n* = 8 for each group) to explore any “cross-talk” between the circulation and a potential target tissue, as accumulating evidence demonstrates that c-miRs can be transferred to and taken up by recipient cells and tissues where they can regulate their specific targets (Fabbri et al., 2012; Russell and Lamon, 2015).

PCR arrays were run using a miScript SYBR Green PCR Kit (339346; Qiagen, Australia). A quantification cycle above 40 in >50% of the samples was considered as absence of expression of the miRNA. Moreover, samples that exhibited low abundance (relative threshold cycle above 40) were excluded from analysis as indicated in Table 3. The array also contained an inter-plate calibrator, along with UniSp3 and UniSp6 RNA Spike-in controls, RNU1A1 and SNORD44. Due to low circulating expression of SNORD44, c-miR expression was normalized to the geometric mean of UniSp3, UniSp6, and RNU1A1. In contrast, AT miRNAs were normalized to the geometric mean of SNORD44, UniSp3, UniSp6, and RNU1A1. The 2^ΔΔ^CT method of relative quantification was used to calculate the relative abundance of miRNAs in plasma and AT (Livak and Schmittgen, 2001).

### Blood Biochemistry and Insulin Sensitivity

Plasma glucose and serum insulin concentrations and Homeostatic Model Assessment for Insulin Resistance (HOMA-IR) were determined for the case-control study (PCOS and Non-PCOS). Plasma glucose concentration was measured using a Roche Moduclar P (Roche, Switzerland), while serum insulin concentration was measured in duplicates using an enzyme-linked immunosorbent assay (ELISA; IBL-International, Germany). HOMA-IR was used as a method for quantifying insulin resistance; fasting serum (μU/mL) × fasting plasma glucose (mmol/L) divided by 22.5 (Matthews et al., 1985).

### Statistics

Descriptive statistics are presented as means ± SD. Group means for PCOS vs Non-PCOS were compared by Student’s two-tailed *t*-test for independent samples. We used linear mixed models with participant as random factor, and with two dummy variables to uniquely identify the two exercise groups (LV-HIT and HV-HIT) and their interactions, with time as fixed factors. We adjusted for the baseline values for the outcome variables as recommended by Twisk et al. (2018). In this model, the coefficients for the interaction terms give the estimated exercise training effects in LV-HIT and HV-HIT compared to the Non-Ex group. Paired *t*-tests were used to test for differences in miRNA expression in AT depots (gluteal versus abdominal) at baseline. To test if miRNA expression in the two AT depots would respond in distinct ways to the two HIT protocols, changes in miRNA expression were calculated by using post-intervention minus pre-intervention values (corresponding to the ΔmiR expression in Table 6). We used linear mixed models as detailed but with the two exercise groups’ interaction with AT depots as fixed factors. Due to previously observed abnormal expression of miRNAs in subcutaneous AT in insulin resistant women regardless of PCOS status, we also analyzed the gluteal and abdominal subcutaneous AT miRNA expression based on insulin resistance. Insulin resistance was estimated as HOMA-IR, with similar cut-off points as previously reported (Chuang et al., 2015); HOMA-IR value <2.5 was considered normal whereas HOMA-IR ≥ 2.5 indicated insulin resistance. Group means for insulin resistant and non-insulin resistant women were compared by Student’s two-tailed *t*-test for independent samples. Normality of residuals was evaluated using visual inspection of Q-Q plots and logarithmic transformation (log10) of the dependent variable was performed when necessary to obtain normality. Due to multiple hypotheses, we considered *P-values* <0.01 as statistically significant. Pearson correlation between miRNAs different between groups (PCOS and Non-PCOS) and baseline VO_2_peak, BMI, fat percentage or waist/hip ratio was calculated using GraphPad Prism version 8.1.2 (GraphPad Software, United States). All other analyses were carried out using SPSS version 25.0 (SPSS Inc., United States).

## Results

### Characteristics: PCOS vs Non-PCOS Women

Characteristics of the participants included in the case-control comparison are presented in Table 1. Apart from greater waist/hip ratio in women with PCOS (0.90 ± 0.06 versus 0.83 ± 0.05, *P* = 0.005), there were no differences between groups (Table 1). There was no difference in HOMA-IR between the groups, although nine of the women with PCOS versus five women without PCOS had HOMA-IR ≥ 2.5 (Table 1).

**TABLE 1:** Baseline characteristics of PCOS and Non-PCOS women.

	**Non-PCOS**	**PCOS**	***P-value***
*n*	12	12	
Age (years)	30 ± 7	30 ± 7	0.98
Body weight (kg)	83.0 ± 18.5	85.2 ± 19.7	0.78
BMI (kg/m^2^)	29.3 ± 5.8	29.8 ± 6.5	0.85
Body fat percentage (%)	36.4 ± 9.2	36.8 ± 9.0	0.91
Waist circumference (cm)	94 ± 14	102 ± 14	0.16
Hip circumference (cm)	113 ± 13	113 ± 11	0.99
Waist/Hip ratio	0.83 ± 0.05	0.90 ± 0.06	**0.005**
VO_2_peak (L/min)	2.83 ± 0.38	2.73 ± 0.38	0.54
VO_2_peak (mL/min/kg)	34.9 ± 7.0	33.2 ± 6.1	0.53
Glucose (mmol/L)	4.9 ± 0.4	5.0 ± 0.6	0.52
Insulin (μU/mL)	14.2 ± 10.6	15.7 ± 6.9	0.71
HOMA-IR	3.1 ± 2.3	3.6 ± 1.8	0.58

*Values are mean ± SD. PCOS, polycystic ovary syndrome; BMI, body mass index; VO_2_peak, peak oxygen uptake; HOMA-IR, homeostatic model assessment of insulin resistance. In the Non-PCOS group, not enough blood was collected for analyzing insulin concentration, so *n* = 11 for insulin concentration and HOMA-IR in Non-PCOS. The meaning of a bold value is that the P-value is statistically significant.*

### Physiological Responses to High-Intensity Interval Training

There were no changes in body weight, BMI, body fat percentage, waist, and hip circumference or waist/hip ratio for either LV-HIT or HV-HIT post intervention (Table 2). Although not significant, it is worth noting that the mean fat percentage decreased by 1.4% in the Non-Ex group. This is not a systemic decrease in fat percentage in the Non-Ex group but a result of two participants in this group decreasing by 6% in fat percentage. Following 16 weeks of HIT, absolute, but not relative, VO_2_peak increased by 9% (*P* = 0.008) in HV-HIT (Table 2).

**TABLE 2:** Participant characteristics before and after 16 weeks of high-intensity interval training.

	**Pre**		**Post**		**Difference (time × group)**		
	***n***	**Mean ± SD**	***n***	**Mean ± SD**	**Estimate**	**95% CI**	***P-value***
**Age (years)**							
Non-Ex	15	28 ± 5					
LV-HIT	13	31 ± 5					
HV-HIT	14	30 ± 5					
**Body weight (kg)**							
Non-Ex	15	86.2 ± 20.1	15	84.1 ± 19.3			
LV-HIT	13	84.6 ± 19.1	13	81.8 ± 20.2	–0.75	−5.28 to 3.78	0.74
HV-HIT	14	92.6 ± 22.6	14	91.9 ± 23.4	1.66	−2.78 to 6.11	0.45
**BMI (kg/m^2^)**							
Non-Ex	15	31.4 ± 6.9	15	30.6 ± 6.5			
LV-HIT	13	28.9 ± 6.6	13	28.8 ± 6.7	0.65	−0.08 to 1.38	0.08
HV-HIT	14	33.1 ± 7.4	14	32.8 ± 7.6	0.59	−0.13 to 1.30	0.11
**Body fat percentage (%)**							
Non-Ex	15	41.0 ± 8.3	15	39.6 ± 8.2			
LV-HIT	13	35.5 ± 10.7	13	35.3 ± 10.4	0.98	−0.75 to 2.72	0.26
HV-HIT	14	41.8 ± 7.9	14	41.2 ± 9.0	0.82	−0.88 to 2.52	0.34
**Waist circumference (cm)**							
Non-Ex	15	101 ± 16	15	99 ± 16			
LV-HIT	13	98 ± 17	12	95 ± 18	–0.63	−5.83 to 4.56	0.81
HV-HIT	14	110 ± 17	14	105 ± 16	–2.46	−7.44 to 2.53	0.33
**Hip circumference (cm)**							
Non-Ex	15	115 ± 14	15	114 ± 12			
HV-HIT	13	112 ± 14	12	111 ± 12	0.01	−3.32 to 3.35	0.99
LV-HIT	14	117 ± 14	14	115 ± 15	–1.22	−4.42 to 1.98	0.45
**Waist/Hip ratio**							
Non-Ex	15	0.88 ± 0.07	15	0.87 ± 0.09			
LV-HIT	13	0.87 ± 0.07	12	0.85 ± 0.09	–0.005	−0.05 to 0.04	0.81
HV-HIT	14	0.94 ± 0.06	14	0.91 ± 0.08	0.01	−0.03 to 0.05	0.65
**VO_2peak_ (L/min)**							
Non-Ex	15	2.73 ± 0.49	15	2.63 ± 0.45			
LV-HIT	13	2.85 ± 0.29	13	2.85 ± 0.39	0.13	−0.07 to 0.32	0.20
HV-HIT	14	2.81 ± 0.34	13	2.94 ± 0.38	0.27	0.07 to 0.46	**0.008**
**VO_2peak_ (mL/kg/min)**							
Non-Ex	15	32.2 ± 6.9	15	32.5 ± 7.5			
LV-HIT	13	34.4 ± 8.6	13	35.5 ± 8.0	0.88	−1.45 to 3.20	0.45
HV-HIT	14	31.7 ± 6.5	13	34.6 ± 8.0	1.97	−0.36 to 4.30	0.10

*The difference (time × group) is the exercise training effect in LV-HIT and HV-HIT compared to the Non-Ex group. n, number of participants; SD, standard deviations; CI, confidence interval; Non-Ex, non-exercising group; LV-HIT, 10 × 1 min exercise group; HV-HIT, 4 × 4 min exercise group; BMI, body mass index; VO_2peak_, peak oxygen uptake. The meaning of a bold value is that the P-value is statistically significant.*

### miRNA Expression Profile in PCOS vs Non-PCOS

**FIGURE 2:**
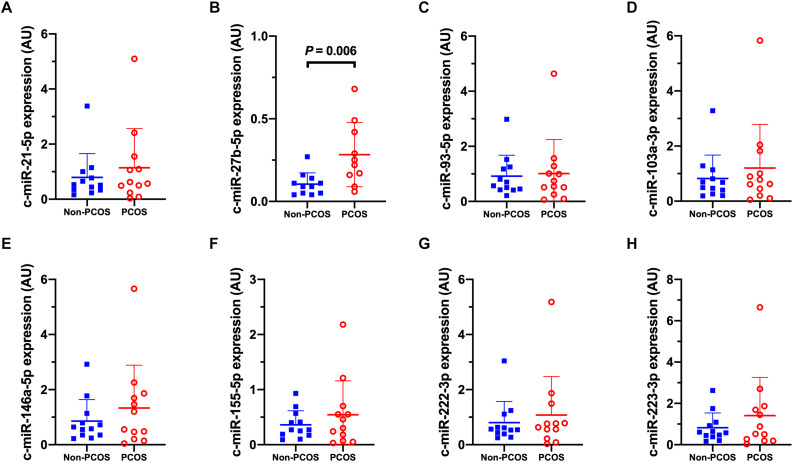
Circulatory microRNA expression patterns. **(A)** c-miR-21-5p, **(B)** c-miR-27b-5p, **(C)** c-miR-93-5p, **(D)** c-miR-103a-3p, **(E)** c-miR-146a-5p, **(F)** c-miR-155-5p, **(G)** c-miR-222-3p, **(H)** c-miR-223-3p abundance in women with (PCOS; open, red circles) and without PCOS (Non-PCOS; blue squares). Values are arbitrary units expressed relative to the geometric mean of UniSp3, UniSp6 and RNU1A1. Individual data with group means and SD are displayed. PCOS, polycystic ovary syndrome; c, circulating; miR, microRNA; AU, arbitrary units.

**FIGURE 3:**
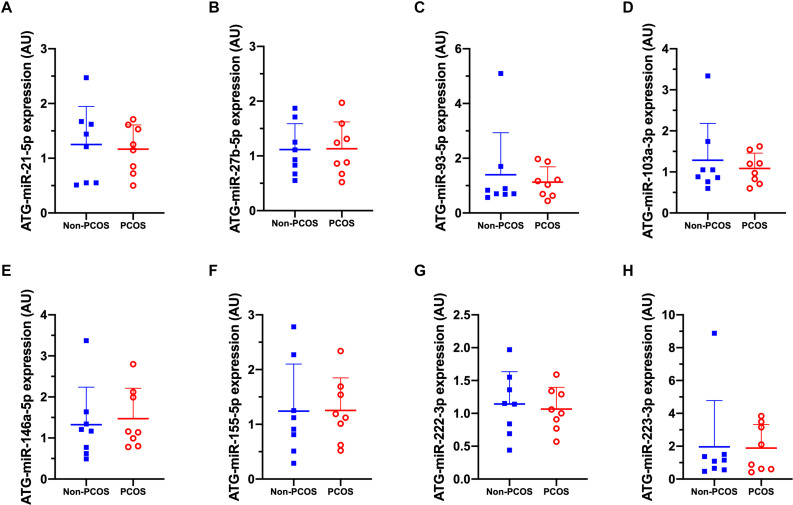
Gluteal adipose tissue (ATG) microRNA expression patterns. **(A)** ATG-miR-21-5p, **(B)** ATG-miR-27b-5p, **(C)** ATG-miR-93-5p, **(D)** ATG-miR-103a-3p, **(E)** ATG-miR-146a-5p, **(F)** ATG-miR-155-5p, **(G)** ATG-miR-222-3p, **(H)** ATG-miR-223-3p abundance in women with (PCOS; open, red circles) and without PCOS (Non-PCOS; blue squares). Values are arbitrary units expressed relative to the geometric mean of SNORD44, UniSp3, UniSp6, and RNU1A1. Individual data with group means and SD are displayed. PCOS, polycystic ovary syndrome; ATG, gluteal adipose tissue; miR, microRNA; AU, arbitrary units.

**FIGURE 4:**
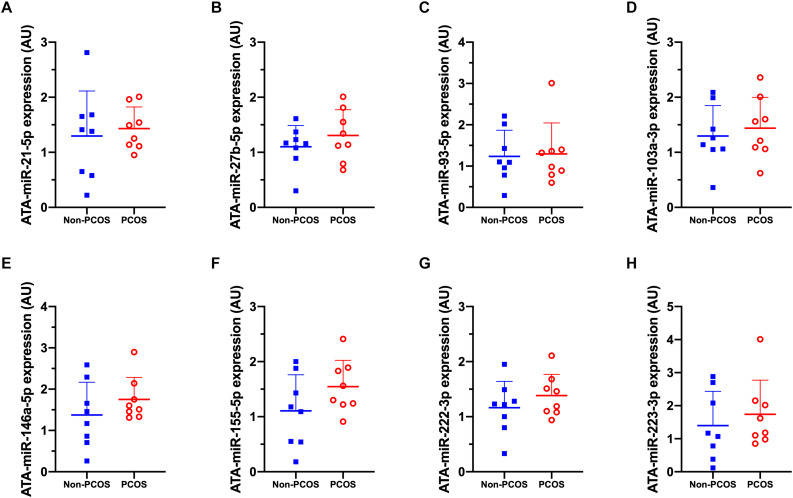
Abdominal adipose tissue (ATA) microRNA expression patterns. **(A)** ATA-miR-21-5p, **(B)** ATA-miR-27b-5p, **(C)** ATA-miR-93-5p, **(D)** ATA-miR-103a-3p, **(E)** ATA-miR-146a-5p, **(F)** ATA-miR-155-5p, **(G)** ATA-miR-222-3p, **(H)** ATA-miR-223-3p abundance in women with (PCOS; open, red circles) and without PCOS (Non-PCOS; blue squares). Values are arbitrary units expressed relative to the geometric mean of SNORD44, UniSp3, UniSp6, and RNU1A1. Individual data with group means and SD are displayed. PCOS, polycystic ovary syndrome; ATA, abdominal adipose tissue; miR, microRNA; AU, arbitrary units.

Basal expression of circulating miRNA-27b (c-miR-27b) was higher in women with PCOS compared to Non-PCOS (1.8-fold, *P* = 0.006; Figure 2B), with no significant differences in the expression of c-miR-21, −93, −103a, −146a, −155, −222, and −223 (Figures 2A–H). No differences in basal gluteal and abdominal AT miRNA expression between women with PCOS and non-PCOS were observed (Figures 3A–H, 4A–H). No correlations were observed between basal c-miR-27b expression and baseline VO_2_peak, BMI, fat percentage, or waist/hip ratio (data not shown).

### Effect of HIT on miRNA Expression Profile

The expression of the selected miRNAs in the circulation, gluteal and abdominal AT before and after 16 weeks of HIT in women with PCOS are summarized in Tables 3–5. In LV-HIT, the expression of c-miR-27b decreased significantly after training (0.5-fold, *P* = 0.007; Table 3), while the expression of c-miR-155 showed a tendency to decline (*P* = 0.06, Table 3). There were no changes in circulating miR-21, -93, -103a, -146a, -155, -222, and -223 in either of the groups. Chronic exercise training did not alter the expression of any of the selected miRNAs in the two AT depots in women with PCOS (Tables 4, 5).

**TABLE 3:** Circulatory miRNA expression before and after 16 weeks of high-intensity interval training.

	**Pre**		**Post**		**Difference (group × time)**
	***n***	**Mean ± SD**	***n***	**Mean ± SD**	***P-value***
**c-miR-21b-5p**					
Non-Ex	14	1.22 ± 0.59	15	2.03 ± 2.83	
LV-HIT	13	1.68 ± 1.84	13	1.61 ± 1.75	0.46
HV-HIT	14	1.61 ± 1.76	14	1.28 ± 0.94	0.30
**c-miR-27b-5p**					
Non-Ex	14	0.73 ± 0.50	15	1.15 ± 1.25	
LV-HIT	12	0.39 ± 0.79	12	0.19 ± 0.20	**0.007**
HV-HIT	11	0.23 ± 0.23	10	0.20 ± 0.10	0.12
**c-miR-93-5p**					
Non-Ex	14	1.07 ± 0.58	15	1.46 ± 1.57	
LV-HIT	13	1.61 ± 1.56	13	1.49 ± 1.34	0.83
HV-HIT	14	1.44 ± 1.46	14	1.83 ± 2.44	0.70
**c-miR-103a-3p**					
Non-Ex	14	1.13 ± 0.63	15	1.73 ± 1.99	
LV-HIT	13	1.75 ± 1.86	13	1.55 ± 1.55	0.59
HV-HIT	14	1.64 ± 1.79	14	1.46 ± 1.33	0.45
**c-miR-146a-5p**					
Non-Ex	14	1.91 ± 0.93	15	2.90 ± 3.46	
LV-HIT	13	1.61 ± 1.71	13	1.45 ± 1.28	0.19
HV-HIT	14	1.58 ± 1.72	14	1.33 ± 1.05	0.10
**c-miR-155-5p**					
Non-Ex	14	0.85 ± 0.53	15	1.25 ± 1.41	
LV-HIT	12	0.94 ± 0.92	13	0.69 ± 0.82	0.06
HV-HIT	14	0.70 ± 0.76	13	0.58 ± 0.39	0.37
**c-miR-222-3p**					
Non-Ex	14	0.95 ± 0.45	15	1.33 ± 1.27	
LV-HIT	13	1.56 ± 1.61	13	1.33 ± 1.30	0.59
HV-HIT	14	1.51 ± 1.59	14	1.39 ± 1.13	0.54
**c-miR-223-3p**					
Non-Ex	14	2.03 ± 1.17	15	3.16 ± 3.80	
LV-HIT	13	1.64 ± 1.86	13	1.55 ± 1.55	0.17
HV-HIT	14	1.69 ± 2.03	14	1.41 ± 1.03	0.14

*The difference (time × group) is the exercise training effect in LV-HIT and HV-HIT compared to the Non-Ex group. n, number of participants; SD, standard deviations; Non-Ex, non-exercising group; LV-HIT, 10 × 1 min exercise group; HV-HIT, 4 × 4 min exercise group; c, circulating; miR, microRNA. The meaning of a bold value is that the P-value is statistically significant.*

**TABLE 4:** Gluteal adipose tissue miRNA expression before and after 16 weeks of high-intensity interval training.

	**Pre**		**Post**		**Difference (group × time)**
	***n***	**Mean ± SD**	***n***	**Mean ± SD**	***P-value***
**ATG-miR-21-5p**					
Non-Ex	8	1.07 ± 0.42	8	2.18 ± 3.09	
LV-HIT	8	1.07 ± 0.39	8	1.03 ± 0.54	0.25
HV-HIT	8	1.41 ± 1.25	8	1.49 ± 1.52	0.48
**ATG-miR-27b-5p**					
Non-Ex	8	1.07 ± 0.41	8	1.43 ± 1.46	
LV-HIT	8	1.12 ± 0.55	8	0.96 ± 0.25	0.53
HV-HIT	8	1.02 ± 0.56	8	1.29 ± 1.28	0.69
**ATG-miR-93-5p**					
Non-Ex	8	1.17 ± 0.78	8	1.94 ± 2.92	
LV-HIT	8	1.09 ± 0.47	8	0.99 ± 0.58	0.41
HV-HIT	8	0.91 ± 0.54	8	0.89 ± 0.59	0.19
**ATG-miR-103a-3p**					
Non-Ex	8	1.09 ± 0.52	8	2.12 ± 3.25	
LV-HIT	8	1.07 ± 0.36	8	0.97 ± 0.47	0.18
HV-HIT	8	0.95 ± 0.37	8	1.05 ± 0.69	0.15
**ATG-miR-146a-5p**					
Non-Ex	8	1.03 ± 0.30	8	2.01 ± 2.91	
LV-HIT	8	1.19 ± 0.79	8	0.94 ± 0.64	0.20
HV-HIT	8	1.72 ± 1.73	8	1.52 ± 1.24	0.89
**ATG-miR-155-5p**					
Non-Ex	8	1.04 ± 0.33	8	2.79 ± 4.38	
LV-HIT	8	1.10 ± 0.56	8	1.13 ± 0.69	0.19
HV-HIT	8	1.47 ± 1.37	8	1.47 ± 1.22	0.43
**ATG-miR-222-3p**					
Non-Ex	8	1.06 ± 0.41	8	2.10 ± 3.13	
LV-HIT	8	1.07 ± 0.41	8	0.95 ± 0.35	0.16
HV-HIT	8	1.02 ± 0.55	8	1.06 ± 0.65	0.18
**ATG-miR-223-3p**					
Non-Ex	8	1.63 ± 2.03	8	1.87 ± 2.60	
LV-HIT	8	1.46 ± 1.40	8	1.21 ± 1.37	0.39
HV-HIT	8	1.31 ± 1.06	8	1.11 ± 0.79	0.48

*The difference (time × group) is the exercise training effect in LV-HIT and HV-HIT compared to the Non-Ex group. n, number of participants; SD, standard deviations; Non-Ex, non-exercising group; LV-HIT, 10 × 1 min exercise group; HV-HIT, 4 × 4 min exercise group; ATG, gluteal adipose tissue; miR, microRNA.*

**TABLE 5:** Abdominal adipose tissue miRNA expression before and after 16 weeks of high-intensity interval training.

	**Pre**		**Post**		**Difference (group × time)**
	***n***	**Mean ± SD**	***n***	**Mean ± SD**	***P-value***
**ATA-miR-21-5p**					
Non-Ex	8	1.18 ± 0.76	8	0.86 ± 0.39	
LV-HIT	8	1.34 ± 0.93	8	1.06 ± 0.56	0.57
HV-HIT	8	1.33 ± 0.62	8	1.73 ± 2.00	0.19
**ATA-miR-27b-5p**					
Non-Ex	8	1.05 ± 0.41	8	0.90 ± 0.37	
LV-HIT	8	1.23 ± 0.71	8	0.87 ± 0.33	0.93
HV-HIT	8	1.01 ± 0.42	8	1.01 ± 0.66	0.75
**ATA-miR-93-5p**					
Non-Ex	8	1.03 ± 0.28	8	1.10 ± 0.73	
LV-HIT	8	1.33 ± 1.01	8	1.15 ± 0.60	0.67
HV-HIT	8	1.08 ± 0.28	8	1.60 ± 1.37	0.33
**ATA-miR-103a-3p**					
Non-Ex	8	1.04 ± 0.31	8	0.96 ± 0.34	
LV-HIT	8	1.19 ± 0.67	8	1.18 ± 0.33	0.26
HV-HIT	8	1.23 ± 0.40	8	1.38 ± 0.66	0.15
**ATA-miR-146a-5p**					
Non-Ex	8	1.10 ± 0.50	8	0.94 ± 0.39	
LV-HIT	8	1.44 ± 1.10	8	1.38 ± 1.10	0.52
HV-HIT	8	1.47 ± 0.74	8	2.16 ± 2.00	0.07
**ATA-miR-155-5p**					
Non-Ex	8	1.14 ± 0.57	8	0.80 ± 0.29	
LV-HIT	8	1.38 ± 0.98	8	1.36 ± 1.01	0.22
HV-HIT	8	1.40 ± 0.65	8	1.56 ± 0.87	0.07
**ATA-miR-222-3p**					
Non-Ex	8	1.03 ± 0.27	8	1.05 ± 0.42	
LV-HIT	8	1.19 ± 0.55	8	1.04 ± 0.31	0.89
HV-HIT	8	1.15 ± 0.48	8	1.27 ± 0.78	0.49
**ATA-miR-223-3p**					
Non-Ex	8	1.11 ± 0.60	8	1.50 ± 1.22	
LV-HIT	8	1.75 ± 1.82	8	2.54 ± 3.60	0.73
HV-HIT	8	1.83 ± 1.16	8	1.79 ± 1.58	0.91

*The difference (time × group) is the exercise training effect in LV-HIT and HV-HIT compared to the Non-Ex group. n, number of participants; SD, standard deviations; Non-Ex, non-exercising group; LV-HIT, 10 × 1 min exercise group; HV-HIT, 4 × 4 min exercise group; ATA, abdominal adipose tissue; miR, microRNA.*

At baseline, none of the selected miRNAs were differentially expressed in gluteal compared to abdominal AT (data not shown). Furthermore, there were no differences in how the selected miRNAs changed in abdominal versus gluteal AT in response to either of the two HIT interventions (Table 6).

**TABLE 6:** Adipose tissue depots differences (gluteal versus abdominal) in miRNA expression response to high-intensity interval training.

	**Gluteal AT**		**Abdominal AT**		**Difference (group × AT depots)**
	***n***	**Mean ± SD**	***n***	**Mean ± SD**	***P-value***
**ΔmiR-21-5p**					
Non-Ex	8	1.11 ± 3.13	8	−0.32 ± 0.52	
LV-HIT	8	−0.04 ± 0.52	8	−0.28 ± 1.01	0.96
HV-HIT	8	0.08 ± 2.19	8	0.40 ± 1.77	0.73
**ΔmiR-27b-5p**					
Non-Ex	8	0.36 ± 1.72	8	−0.15 ± 0.43	
LV-HIT	8	−0.16 ± 0.57	8	−0.37 ± 0.68	0.80
HV-HIT	8	0.27 ± 1.50	8	0.00 ± 0.79	0.78
**ΔmiR-93-5p**					
Non-Ex	8	0.77 ± 0.07	8	0.06 ± 0.57	
LV-HIT	8	−0.10 ± 0.87	8	−0.18 ± 1.26	0.98
HV-HIT	8	−0.02 ± 0.88	8	0.52 ± 1.27	0.77
**ΔmiR-103a-3p**					
Non-Ex	8	1.03 ± 3.35	8	−0.08 ± 0.32	
LV-HIT	8	−0.09 ± 0.61	8	−0.01 ± 0.86	0.87
HV-HIT	8	0.10 ± 0.87	8	0.15 ± 0.56	0.95
**ΔmiR-146a-5p**					
Non-Ex	8	0.97 ± 2.86	8	−0.17 ± 0.43	
LV-HIT	8	−0.26 ± 0.67	8	−0.07 ± 1.19	0.97
HV-HIT	8	−0.20 ± 2.34	8	0.68 ± 2.09	0.48
**ΔmiR-155-5p**					
Non-Ex	8	1.75 ± 4.42	8	−0.33 ± 0.49	
LV-HIT	8	0.03 ± 0.78	8	−0.03 ± 1.15	0.63
HV-HIT	8	0.00 ± 2.04	8	0.16 ± 0.91	0.84
**ΔmiR-222-3p**					
Non-Ex	8	1.04 ± 3.24	8	0.02 ± 0.44	
LV-HIT	8	−0.13 ± 0.49	8	−0.15 ± 0.69	0.71
HV-HIT	8	0.04 ± 1.00	8	0.13 ± 0.94	0.92
**ΔmiR-223-3p**					
Non-Ex	8	0.24 ± 1.09	8	0.39 ± 1.17	
LV-HIT	8	−0.25 ± 2.02	8	0.79 ± 2.77	0.53
HV-HIT	8	−0.19 ± 1.46	8	−0.04 ± 1.57	0.52

*n, number of participants; SD, standard deviations; Non-Ex, non-exercising group; LV-HIT, 10 × 1 min exercise group; HV-HIT, 4 × 4 min exercise group; AT, adipose tissue; miR, microRNA. ΔmiR corresponds to the changes in miRNA expression calculated by using post-intervention minus pre-intervention values.*

### Gluteal and Abdominal AT miRNA Expression Profile in Insulin Resistant and Non-insulin Resistant Women

There were no differences in basal gluteal and abdominal AT miRNA expression between insulin resistant and non-insulin resistant women regardless of PCOS status (data not shown). miR-103a tended to be lower in insulin resistant women in gluteal subcutaneous AT (*P* = 0.09) and miR-155 tended to be higher in insulin resistant women in abdominal subcutaneous AT (*P* = 0.08).

## Discussion

We report novel data regarding miRNA expression profiles in women with and without PCOS, along with responses to 16 weeks of HIT undertaken by women with PCOS. We show that basal expression of c-miR-27b was higher in women with PCOS compared to age- and BMI-matched women without PCOS. We also show that 16 weeks of exercise training induced a significant decrease in c-miR-27b in women with PCOS, despite no changes in the expression profile of the other targeted c-miRs. Finally, we show that miRNA expression from the two separate subcutaneous AT sites was unaffected by exercise training. Collectively, our findings provide new data regarding the effects of chronic exercise training and selected miRNAs responses in women with PCOS.

The first important finding from the current investigation was that basal c-miR-27b expression was higher in women with PCOS compared to age- and BMI-matched women without PCOS. miRNA-27b has been implicated in several cellular and metabolic processes including fatty acid metabolism, adipocyte differentiation, substrate metabolism, and inflammation (Fernandez-Valverde et al., 2011; Chen W. et al., 2012; Murri et al., 2013). Murri et al. (2013) reported that women without PCOS with a BMI > 30 kg/m^2^ had lower basal whole blood miR-27b expression compared to women with normal weight (BMI < 25 kg/m^2^), whereas obesity was associated with higher expression of miR-27b compared to normal weight in women with PCOS. These findings, together with our data, indicate divergent expression of basal c-miR-27b in women with and without PCOS. Previous work showed that overexpression of miR-27b attenuated the abundance of regulators of adipogenesis such as peroxisome proliferator-activated receptor-γ (PPAR-γ) and CCAAT/enhancer-binding protein α (C/EBPα) (Lin et al., 2009), with PPAR-γ confirmed as a direct target of miR-27b (Karbiener et al., 2009). Similarly, work in diabetic rats and 3T3-L1 adipocytes revealed elevated expression of the closely related miR-27a within the retroperitoneal fat pad and in the presence of high glucose, respectively (Herrera et al., 2010). While these findings indicate a role for miR-27b in the etiology of adipogenesis and obesity, we found no relationship between basal c-miR-27b expression and BMI, fat percentage or waist/hip ratio, possibly indicating the cellular effects of miR-27b are likely to be confined more locally to within particular AT depots rather than whole body in such adipogenic models (Karbiener et al., 2009; Herrera et al., 2010).

Accumulating evidence demonstrates the capacity of vesicle-carrying miRNAs within the circulatory system to be released into the cytosol of recipient cells where they can regulate specific mRNA targets (Mittelbrunn et al., 2011; Fabbri et al., 2012). As such, we investigated miRNA expression within the subcutaneous abdominal and gluteal AT depots of women with PCOS. Somewhat surprisingly, and in contrast to one of our original hypotheses, we found no differences in miRNA expression signatures between women with and without PCOS in either of the subcutaneous adipose sites. Previously, Chuang et al. (2015) reported significantly higher miR-223 expression in subcutaneous abdominal AT in insulin resistant women regardless of PCOS status. However, when data from insulin resistant (HOMA-IR ≥ 2.5) and insulin sensitive (HOMA-IR ≤ 2.5) women were pooled, there was no difference in miR-223 expression between women with and without PCOS (Chuang et al., 2015). Similarly, the expression patterns of other subcutaneous abdominal AT miRNAs have been reported to be similar between women with and without PCOS, but to be different between insulin resistant (HOMA-IR ≥ 2.5) and insulin sensitive (HOMA-IR < 2.5) women (Wu et al., 2014). We found no differences in either gluteal or abdominal AT miRNA expression profiles between insulin resistant and non-insulin resistant women (regardless of PCOS status). Our ability to detect significant differences between these cohorts may, in part, be due to a lack of statistical power because of low subject numbers in the present study.

Despite women with PCOS having significantly higher basal expression of c-miR-27b, there were no differences in the tissue abundance of this miRNA in either subcutaneous abdominal or gluteal AT. This “disconnect” in miRNA expression between the circulatory system and AT could be due to several reasons. First, the origin of c-miR-27b is unknown. Second, the subcutaneous AT depots may lack the specific membrane transporters to import this miRNA from the circulation (Icli and Feinberg, 2017). Third, temporal differences in miRNA expression are likely to exist between different body compartments (i.e., in the systemic circulation versus various tissues/organs). Future work will be required to underpin the mechanistic basis of miRNA “cross-talk” between the circulation and target tissues, and may provide important information linking PCOS and adipogenesis.

Another finding from the current investigation was the decrease in c-miR-27b expression in women with PCOS after 16 weeks of low-, but not high-volume, HIT. It is difficult to explain such a divergent response, especially as only the high-volume HIT protocol was associated with a significant increase in cardiorespiratory fitness. While little is known about c-miR-27b expression patterns following exercise training, Barber et al. (2019) recently reported that 20 weeks of moderate-intensity endurance training in middle aged men and non-PCOS women was associated with a two-fold increase in c-miR-27b (Barber et al., 2019). The discrepancy between our current work and the findings by Barber et al. (2019) may be explained by differences in exercise modality. HIT versus moderate-intensity continuous endurance training can differentially influence cardiometabolic risk factors including blood pressure, low- and high-density lipoproteins, body weight and insulin sensitivity in patients with cardiometabolic diseases (Weston et al., 2014). Additionally, diverse sampling timepoint (24 h post last training session in the study by Barber et al. (2019) compared to≥48 h in our study) and study participants (middle aged men and women without PCOS compared to women with PCOS in our study) may, in part, help explain these divergent findings. Parr et al. (2016), Denham et al. (2018), Barber et al. (2019), and Da Silva et al. (2019) have previously reported that chronic exercise training programs can significantly modulate c-miRNA expression in healthy individuals and in those with overweight/obesity. The physiological significance of such responses are unclear and difficult to explain due to a variety of different miRNA quantification techniques reported, differences in sampling time points, a range of exercise protocols, and divergent clinical cohorts (Gomes et al., 2015). Time-course studies are needed to span the acute and chronic sampling points for a more comprehensive coverage of miRNA expression profiles.

We found no changes in miRNA expression in either subcutaneous abdominal or gluteal AT after high- or low-volume HIT protocols in women with PCOS. This observation is in agreement with recent data from Tsiloulis et al. (2017) who reported no effect of 6 weeks of endurance exercise training (four supervised sessions/week; three moderate intensity sessions; and one HIT session) on the expression of 526 miRNAs in abdominal and gluteal AT in overweight males. Collectively, these findings indicate miRNAs are stably expressed in both abdominal and gluteal AT and are largely unaltered in response to exercise training protocols lasting up to several months. Furthermore, at baseline, we found no difference in expression of the eight selected miRNAs in basal gluteal versus abdominal AT. Our findings are largely supported by the results of Tsiloulis et al. (2017), who reported only four out of the 526 identified miRNAs to be differentially expressed between gluteal and abdominal adipocytes in overweight males. In addition, we found no differentially expressed miRNAs between the AT depots following exercise training. Other studies have reported that AT from different depots express unique molecular, cellular, and metabolic properties and respond in distinct ways to exercise and nutritional challenges (Tan et al., 2004; You et al., 2014). However, this does not seem to be the case for selected miRNA expression.

A major strength of the present study is the concurrent investigation of miRNA expression patterns in the circulation and abdominal and gluteal AT. Some miRNAs exert specificity in tissue expression within the body and can either be released or taken up by these tissues from the bloodstream (Russell and Lamon, 2015). Thus, investigation of targeted miRNAs from both the circulation and a “target” tissue allowed us to compare expression patterns between these tissues. We also acknowledge study limitations: we used a targeted approach to selectively investigate a small number of miRNAs and therefore may have missed changes in expression patterns of other miRNAs. Finally, larger subject numbers would have conferred greater statistical power to detect small, but potential differences in miRNA expression.

In conclusion, we report that the basal expression of circulating miR-27b was higher in women with PCOS compared to women without PCOS. We were unable to detect any differences in the eight targeted miRNAs in subcutaneous abdominal and gluteal AT. While 16 weeks of low- but not high-volume HIT altered the expression of c-miR-27b in women with PCOS, the chronic exercise training protocols employed in this investigation did not induce alterations in the expression of the other targeted miRNAs within the circulation or the miRNAs in subcutaneous abdominal and gluteal AT in women with PCOS. Further studies are required to ascertain miR-27b’s role and association with obesity, inflammation and adipogenesis in PCOS.

## Data Availability Statement

The raw data supporting the conclusions of this article will be made available by the authors, without undue reservation.

## Ethics Statement

The studies involving human participants were reviewed and approved by The Regional Committee for Medical and Health Research Ethics in Central Norway (REK-midt 2015/468 and 2016/545) and the Australian Catholic University Human Research Ethics Committee (2017-260H). The patients/participants provided their written informed consent to participate in this study.

## Author Contributions

SL and DC drafted the manuscript, performed the analyses, and analyzed the data. SL and SLy performed statistical analyses. SL, IK, DC, EV, JH, and TM were responsible for study conception and design. SL, IK, EP, and TM coordinated the study at the two sites, performed measurements on test-days, and supervised the exercise training. All authors provided feedback and approved the final manuscript.

## Conflict of Interest

The authors declare that the research was conducted in the absence of any commercial or financial relationships that could be construed as a potential conflict of interest.
